# Feeding a high-grain diet reduces the percentage of LPS clearance and enhances immune gene expression in goat liver

**DOI:** 10.1186/s12917-015-0376-y

**Published:** 2015-03-18

**Authors:** Guangjun Chang, Kai Zhang, Tianle Xu, Di Jin, Hans-Martin Seyfert, Xiangzhen Shen, Su Zhuang

**Affiliations:** College of Veterinary Medicine, Nanjing Agricultural University, Nanjing, 210095 PR China; College of Animal Science and Technology, Nanjing Agricultural University, Nanjing, 210095 PR China; Leibniz Institute for Farm Animal Biology, Wilhelm-Stahl-Allee 2, 18196 Dummerstorf, Germany

**Keywords:** High grain diet, Lipopolysaccharide, Immune gene expression, Liver

## Abstract

**Background:**

The effects of feeding a high-grain (HG) diet on lipopolysaccharide (LPS) clearance and innate immune defence responses in the liver remain unclear. Therefore, we conducted the present study in which twelve female goats were randomly assigned to either a treatment group fed a HG diet (60% grain, n = 6) or a control group fed a low grain diet (LG; 40% grain, n = 6) for 6 weeks. Catheters were installed in the mesenteric, portal and hepatic veins, as well as one femoral artery of the goats, for determining blood flow and net clearance rate of LPS in the liver. Plasma and tissue samples were collected in the week 6 for analyzing pro-inflammatory cytokines, acute phase protein and biochemical parameters, as well as expression of genes involved in immune response.

**Result:**

HG diet feeding increased blood flow and LPS concentration in the portal vein, hepatic vein and artery. Hepatic net LPS clearance showed that HG diet feeding elevated the rate of hepatic LPS clearance, but decreased the percentage of removed LPS accounting for the total entry of LPS into the liver. Our results demonstrated that the feeding of HG diet increased plasma concentrations of pro-inflammatory cytokines and acute phase proteins and triggered a systemic inflammatory response. In addition, peripheral blood plasma concentrations of alanine aminotransferase, alkaline phosphatase and total bilirubin were increased in the HG group compared to the LG group. This indicated that the impairment of hepatocytes occurred after 6 weeks of HG diet feeding. The expression of genes involved in immune response and Toll-like receptor (TLR)4 protein in the liver was up-regulated in the HG group compared to the LG group, indicating that increased entry of LPS enhanced hepatic immune defence responses and contributed to hepatic inflammatory responses.

**Conclusion:**

These results provide insight into the capacity of the liver to clear LPS. The increased entry of LPS into liver enhanced hepatic immune defence responses, thereby elevated the rate of LPS clearance. However, the reduction of the percentage of hepatic LPS clearance could be due to the formation of hepatocyte lesion during HG diet feeding.

## Background

Ruminants are fed a high grain diet to support high milk yields or induce rapid weight gain in modern animal husbandry. However, the long-term consumption of high grain diet is harmful to the health of ruminants. It has been reported that the feeding of high grain diet resulted in reduced pH values and increased lipopolysaccharide (LPS) concentrations in the digestive tract [1-3]. These changes cause local inflammatory responses of digestive tract and injury to the gastrointestinal barrier [4-6], which facilitates the translocation of LPS from the digestive tract into the bloodstream [7,8].

Many studies have demonstrated that increased circulating LPS cause a systemic inflammatory response [8-10]. Acute phase proteins (APPs), such as serum amyloid A (SAA), haptoglobin (Hp) and LPS-binding protein (LBP), are biomarkers for the diagnosis of inflammation and infection [11]. An increase of SAA and Hp concentrations in the peripheral blood of cows fed a high proportion of grain diet showed that a systemic inflammatory response was activated [8]. The concentration of LBP in peripheral blood is an important indicator for systemic inflammation caused by circulating LPS. An increase of plasma LBP concentration in a grain-induced subacute ruminal acidosis (SARA) experimental model indicated that LPS translocated from the digestive tract into the bloodstream to elicit a systemic inflammatory response [1]. In addition, the increased levels of circulating LPS also can elevated the concentration of blood pro-inflammatory cytokines (interleukin (IL)-1, IL-6 and tumour necrosis factor (TNF)-α). A recent study showed that the concentration of pro-inflammatory cytokines was significantly increased in an LPS intra-mammary infusion experiment [12]. The feeding of a high concentrate corn straw diet also resulted in increased pro-inflammatory cytokines in mammary arteries caused by the translocation of LPS from the digestive tract into the bloodstream [13].

Experimentally-induced endotoxic shock demonstrated that the liver is a major contributor of inflammatory cytokines in the bloodstream [14]. Circulating LPS entering the liver is recognized by Toll-like receptor (TLR) 4, which is expressed on the surface of Kupffer cells (liver macrophages) and other immune cells, to orchestrate the synthesis and secretion of cytokines and chemokines [15-17]. Subsequently, those cytokines can activate intracellular signalling pathways between immune cells and hepatocytes to regulate hepatic APP synthesis [18]. The synthesis of SAA and Hp was enhanced in primary bovine hepatocytes in response to stimulation with recombinant human pro-inflammatory cytokines [19]. Intravenous or intramammary infusion of LPS is frequently used in an *Escherichia coli-*induced mastitis model [12,20,21], where expression of cytokines and APPs was significantly increased in the liver of experimental animals. However, the production of excessive cytokines in the liver can cause functionally- impaired hepatocytes [22,23].

It is well known that the liver is the main site for clearance of circulating LPS [24,25], and these hepatic removing mechanisms have been well documented [26,27]. The significant increase in LPS concentrations in the bloodstream during feeding with high grain diet has received increasing attentions [1,28]. However, few investigations have reported on the rate of hepatic LPS clearance and the percentage of removed LPS accounting for the total entry of LPS into liver. Furthermore, little information is available regarding hepatic immune responses induced by the increased entry of LPS into the liver during the feeding of high grain diet. Therefore, we hypothesized that feeding a high grain diet to goats resulted in the increased entry of LPS into liver, thereby enhanced hepatic immune defence response heightening the rate of hepatic LPS clearance.

## Methods

The experimental design and sampling procedures were approved by the Nanjing Agricultural University Institutional Animal Care and Use Committee before the beginning of this experiment.

### Animals, diets and experimental design

Goats were housed in individual metabolic cage in the Centre of Experimental Animal at Nanjing Agricultural University (Nanjing, China). Twelve non-lactating and non-pregnant GuanZhong dairy goats (body weight 40.56 ± 1.34 kg, mean ± SEM) aged 2–3 years were used in experiments. All goats received a low grain diet (LG; forage: concentrate = 6:4) for weeks before the start of the formal experiment as an adaption period to obtain a similar metabolic status in all individuals. The goats were randomly assigned to two groups: goats were fed an LG diet (*n =* 6) as the control group or fed a high grain diet (HG; forage: concentrate = 4:6; *n* = 6) as the treatment group (Table 1). During the experimental period of six weeks, goats were fed two times daily at 8.30 and 16.30, had free access to fresh water, and the feed amount met or exceeded the animal’s nutritional requirements.

**Table 1:** **Chemical composition and nutrient levels of diets**

**Ingredient**	**Percentage (%) of ingredients (dry matter)**	
	**LG diet**	**HG diet**
Chinese wildrye hay	48.00	32.00
Alfalfa hay	12.00	8.00
Corn	28.78	43.17
Soybean meal	8.45	12.68
limestone	0.77	1.25
Calcium phosphate dibasic	1.10	1.65
Salt	0.40	0.50
Premix ^a^	0.50	0.75
Forage: Concentrate	6:4	4:6
**Nutrient levels, % of dry matter**		
Dry mater, %	88.90	88.60
Net energy, MJ/kg	5.40	5.89
CP, %	12.24	13.45
NDF, %	36.55	27.69
ADF, %	24.04	17.54

^a^Premix provided: 3000, 1250, and 40 IU kg^−1^ of diet of vitamin A, D and E, and 6.25, 62.5, 62.5, 50, 0.25, 0.125, 0.125 mg kg^−1^ of diet of Cu, Fe, Zn, Mn, I, Se, Co, respectively. CP: crude protein; NDF: neutral detergent fibre; ADF: acid detergent fibre.

In the first week of the adaption period, Catheters were administered in the mesenteric, portal and hepatic veins, as well as one femoral artery of the goats, for determining blood flow and net clearance rate of LPS in the liver. According to previous study [29], concentration of arterial content is homogeneous, so we used the femoral artery to substitute for hepatic artery. Animals were looked after for 3 weeks after surgery. Sterilized heparin saline (500 IU/mL, 0.3 mL/time) was used to prevent catheter blocking at 8-hour intervals per day until the end of the experiment.

### Sample collection

On the first 3 days of the 6th week, blood samples were collected from the portal vein, hepatic vein, and femoral artery at 0 h (15 min before feeding), and at 2, 4, 6, and 8 h after feeding and injected into a blank 5 mL heparinised evacuated glass tube. Blood samples from the jugular vein were taken at 0 h (15 min before feeding), and at 4 and 8 h after feeding. Samples were kept on ice until transported to the laboratory. Plasma was harvested by centrifuging heparinised evacuated glass tubes at 1900 × *g* for 15 min. Plasma from the portal vein, hepatic vein and femoral artery were transferred into pyrogen-free glass tubes and stored at −20°C for LPS analysis. Plasma from the jugular vein was preserved in sterilized Eppendorf plastic tubes and stored at −20°C for analysis of APPs, cytokines, and biochemical parameters.

After 2 days of blood sampling, goats were measured the body weight and then administered a continuous infusion of a sterilized aqueous solution (pH 7.4) of *para*-aminohippuric acid (*p*AH, 1% (wt/vol), CAS 94-16-6, from Alfa Aesar China Co., Ltd) into the mesenteric vein. The initial rate of infusion was 4 mL/min for 10 min, and then the infusion rate was kept constant (0.8 mL/min) until the end of sampling. Blood samples were collected from the portal vein, hepatic vein, and femoral artery at 0 h (15 min before feeding), and at 2, 4, 6, and 8 h after feeding and injected into a blank 5 mL heparinised evacuated glass tube. Plasma was harvested by centrifuging heparinised evacuated glass tubes at 1900 × *g* for 15 min and transferred into Eppendorf plastic tubes and stored at −20°C for measurement of *p*AH.

On the last day of the 6th week, liver samples were excised immediately after euthanasia. Small frozen tubes (2 mL) were snap-frozen and stored in liquid nitrogen.

### Measurements of plasma parameters

The concentration of *p*AH in the portal vein, hepatic vein and artery were determined as described previously [30]. In brief, plasma was deproteinised by addition of 0.5 mL/L trichloroacetic acid and then spun down at 1000 × *g* for 15 min. A portion (2 g) of supernatant was mixed with 0.5 g of 1.2 mol/L HCL, heated at 95°C for 65 min without charring, and left to cool at room temperature for 15 min. Then, 0.25 mL of 1% sodium nitrite, 0.25 mL of 0.5% ammonium sulphamate and 0.25 mL of 0.1% N-1-napthyl-ethylene-diamino-dihydrochloride were added in time sequence. After 60 min at room temperature, the absorbance of samples and standards was read at 540 nm in a spectrophotometer (V-5600, METASH, Shanghai, China). The concentration of *p*AH was calculated according to the standard curve.

The concentration of LPS in the plasma of the portal vein, hepatic vein and femoral artery was determined by a chromogenic endpoint assay (CE80545, Chinese Horseshoe Crab Reagent Manufactory Co., Ltd., Xiamen, China) with a minimum detection limit of 0.01 EU/mL. The procedures were performed in accordance with the manufacturer’s instruction, as described by Dong *et al.* [31].

Radioimmunoassay was applied to determine the concentrations of master cytokines, including IL-1β, IL-6 and TNF-α in circulating blood. The concentrations of IL-1β, IL-6 and TNF-α were determined with commercially available human radioimmunoassay kits purchased from Beijing North Institute of Biological Technology. The detected range of radioimmunoassay kits for IL-1β (cat. C09DJB), IL-6 (cat. C12DJB) and TNF-α (cat. C06PJB) were 0.1–8.1 ng/mL, 50–4000 pg/ml and 9–590 fmol/mL, respectively.

APPs such as LBP (cat. BP-E93101, Shanghai Lengton), Hp (cat. ab108856, Shanghai Abcam), and SAA (cat. ab100635, Shanghai Abcam) were detected by enzyme-linked immunosorbent assay (ELISA) kits according to the manufacturer’s instructions. ELISA kits for SAA and Hp were already validated by Dong *et al.* [31]. The assay range of the LBP ELISA kit was 62.5–2000 ng/mL. Plasma samples were diluted until the LBP concentration was in the range of this kit.

The plasma concentrations of alanine aminotransferase (ALT), aspartate aminotransferase (AST), total proteins (T-PRO), albumin (ALB), total bilirubin (T-BIL) and alkaline phosphatase (ALP) were determined using enzymatic colorimetric assay kits on an automatic biochemical analyser (Mindray BS-300, Mindray Medical International Limited, Shenzhen, China).

### RNA extraction and real-time quantitative PCR (RT-qPCR)

RT-qPCR was performed using an ABI 7300 instrument to determine the relative copy numbers of the different mRNA. Liver samples were powdered in a mortar under liquid nitrogen and total RNA was extracted with TRIZOL (Takara) according to the manufacturer’s protocol. For cDNA synthesis, 1.5 μg of total RNA was prepared in reverse transcriptional reaction with oligo(dT) for all mRNAs. After reverse transcription (cat. RR036A, Takara) and cDNA purification (cat. D0033, Beyotime), RT-qPCR was run with gene-specific primer pairs to amplify the target segment of cDNA using the SYBR Premix EX Taq™ kit (DRR420A, Takara). Relative copy numbers of the individual mRNA were calculated from a dilution series of 10^6^ to 10^2^ copies of the respective cDNA subclones. All samples were checked twice from two independent cDNA preparations. All primers used for amplification are listed in Table 2.

**Table 2:** **The list of primers for amplification of RT-qPCR**

**Gene**	**Forward primer**	**Reverse primer**	**Length**
TLR4	CTGAGAACCGAGAGCTGGGAC	GCCTTGAAATGTGTTGTCTTCA	207 bp
TLR2	GCTCAGGTGGAAGCTTTCCAG	GGTGATCTCGTTGTTGGACAG	241 bp
IL-1A	GATGATGACCTGGAAGCCATTG	GCTGAGAATCCTCTTCTGATAC	259 bp
IL-1B	CCGTGATGATGACCTGAGGAG	CAAGACAGGTATAGATTCTTGTC	303 bp
IL-6	CGAAGCTCTCATTAAGCACATC	CCAGGTATATCTGATACTCCAG	241 bp
TNF-α	CAACAGGCCTCTGGTTCAGAC	GGACCTGCGAGTAGATGAGG	209 bp
IL-8	CTGAGAGTTATTGAGAGTGGGC	CAGTACTCAAGGCACTGAAGTAG	259 bp
IL-10	GTGATGCCACAGGCTGAGAAC	GAAGATGTCAAACTCACTCATGG	213 bp
CCL5	CTACACCAGCAGCAAGTGCT	CAAGCTGCTTAGGACAAGAGG	190 bp
CCL20	GAAGCAGCAAGCAGCTTTGAC	GTTCCATTCCAGGGAGCATC	244 bp
SAA3	GACATTCCTCAGGGAAGCTG	CTTCGAATCCTTCCGTACCTG	247 bp
Hp	GGAGTACTCGGTTCGCTATCA	CCATCGTTCATTGATGAGTGTG	280 bp
LBP	GAGCTGTCCACCACCAAGATG	CACACTCAGATCAAATGTACCG	243 bp

### Western blotting

Liver tissue was crushed in a mortar under liquid nitrogen and total protein was extracted with RIPA Lysis Buffer (cat. SN338, Sunshine Biotechnology (Nanjing) Co., Ltd). Protein concentration was determined using the BCA assay (Pierce, Rockford, IL, USA). Fifty μg of protein extracted from each sample was applied to electrophoresis on a 7.5% sodium dodecyl sulphate-polyacrylamide gel electrophoresis (SDS-PAGE) gel, and the separated proteins were transferred onto nitrocellulose membranes (Bio Trace, Pall Co., USA). Western blotting analysis for TLR4 (sc-293072, Santa Cruz Biotechnology Inc., 1:200) was performed with the primary antibody and corresponding HRP-conjugated secondary antibody. β-actin (KC-5A08, Kang Chen Bio-Tech, China, 1:5000) was used as a reference protein for normalization in western blotting analyses. Then the blot was washed and detected by enhanced chemiluminescence (ECL) using the LumiGlo substrate (Super Signal West Pico Trial Kit, Pierce, USA). ECL signals were recorded by an imaging system (Bio-Rad, USA) and analyzed with Quantity One software (Bio-Rad, USA). Gray values of TLR4 protein were presented as fold change relative to the mean value of the control group.

### Calculation and statistical analysis

The calculation of blood flow was previously described by Huntington *et al.* [32] and Wieghart *et al.* [33] as follows:

$$ {F}_P\left(L/h\right)={C}_0I/\left({C}_P-{C}_A\right);{F}_H\left(L/h\right)={C}_0I/\left({C}_H-{C}_A\right); $$

$$ {F}_A={F}_H-{F}_P; $$

where I (L/h) is the rate of initial infusion, C_0_ represents the initial content of *p*AH (mg/L); C_P_, C_H_, C_A_ are *p*AH concentration (mg/L) in plasma of portal vein, hepatic vein and artery, respectively. F_P_, F_H_ and F_A_ represent the mean blood flow in the portal vein, hepatic vein and artery. Net clearance rate and ratio in liver were calculated in accordance with the following equations, modified by Lobley [29] and Galindo [34]:

$$ Net\  clearance\  rate\left(EU/h\right)={F}_P\times {P}_{LPS}+{F}_A\times {A}_{LPS}\hbox{--} {F}_H\times {H}_{LPS}; $$

$$ Net\  clearance\  ratio=\left({F}_P\times {P}_{LPS}+{F}_A\times {A}_{LPS}\hbox{--} {F}_H\times {H}_{LPS}\right)\kern0.1em /\kern0.5em \left({F}_P\times {P}_{LPS}+{F}_A\times {A}_{LPS}\right)\times 100\% $$

where P_LPS_, H_LPS_ and A_LPS_ are the concentrations of LPS in plasma of portal vein, hepatic vein and artery.

Data of blood flow, LPS concentration, LPS clearance, and plasma parameters (cytokines, biochemical parameter, and APPs) were analysed with repeated measures using the MIXED procedure of SAS (SAS version 9.2, SAS Institute Inc.). The effects of diet and time were considered fixed. The effects of goats, diet × goats and diet × time × goats were considered random. Time within diet and goat was considered a repeated measure, and compound symmetry (CS) was used as the type of covariance. Data analysis of the expression of mRNAs encoding selected genes involved in immune response and TLR4 protein was performed using the t-test of paired values. A correlation between TLR4 mRNA and protein was analyzed by the Pearson model. Effects were considered significant when *P* < 0.05.

## Results

### Blood flow of hepatic vein, portal vein and artery

The mean blood flows in the hepatic vein (*P* = 0.005), portal vein (*P* < 0.001) and artery (*P* = 0.024) in the HG group were higher than those in the LG group. The highest blood flow in hepatic vein, portal vein and artery was observed at 4 h after feeding in both groups. Results also showed that blood flow was fastest in the hepatic vein, and slowest in arteries (Table 3). In addition, Body weights of both groups did not change during the experiment and averaged 41.23 ± 0.97 kg (not shown in table).

**Table 3:** **The average blood flows in hepatic vein, portal vein and artery of goats**

**Blood flow (L/h)**	**Diet**		**SED** ^**a**^	**Effect,** ***P*** **-value**		
	**LG**	**HG**		**Diet**	**Time**	**Diet × Time**
Hepatic vein						
0 h	120.04	144.87	10.35	0.005	0.419	0.951
2 h	124.35	151.73				
4 h	128.42	167.84				
6 h	128.64	157.67				
8 h	118.01	148.29				
Portal vein						
0 h	100.96	122.26	7.72	<0.001	0.210	0.879
2 h	105.56	129.75				
4 h	111.57	142.71				
6 h	118.99	133.73				
8 h	103.52	127.88				
Artery						
0 h	19.08	22.61	4.76	0.024	0.726	0.627
2 h	18.79	21.98				
4 h	16.85	25.14				
6 h	9.64	23.94				
8 h	14.49	20.42				

^a^SED: standard error of difference between treatment and control.

### LPS concentration in the hepatic vein, portal vein and artery and LPS clearance in liver

Plasma LPS concentrations in the hepatic vein (*P* = 0.001), portal vein (*P* < 0.001) and artery (*P* < 0.001) in the HG group were increased in comparison to those in the LG group. The concentration of LPS in the portal vein was higher than that in the hepatic vein and artery in both groups. The effect of diet × time (*P* < 0.01) on plasma LPS concentrations in the portal vein showed a significant difference between the LG group and HG group. The effect of sampling time on plasma LPS concentrations in the hepatic vein or artery was not significant (Table 4).

**Table 4:** **The average concentrations of LPS in plasma of hepatic vein, portal vein and artery of goats**

**LPS conc. (EU/mL)**	**Diet**		**SED** ^**a**^	**Effect,** ***P*** **-value**		
	**LG**	**HG**		**Diet**	**Time**	**Diet × Time**
Hepatic vein						
0 h	0.41	1.11	0.07	0.001	0.066	0.419
2 h	0.28	1.09				
4 h	0.48	1.23				
6 h	0.39	1.13				
8 h	0.42	1.01				
Portal vein						
0 h	0.47	1.32	0.05	<0.001	0.672	0.011
2 h	0.45	1.28				
4 h	0.52	1.32				
6 h	0.48	1.35				
8 h	0.59	1.13				
Artery						
0 h	0.36	1.08	0.06	<0.001	0.415	0.451
2 h	0.34	1.09				
4 h	0.43	1.15				
6 h	0.39	1.08				
8 h	0.41	0.97				

^a^SED: standard error of difference between treatment and control.

**Figure 1:**
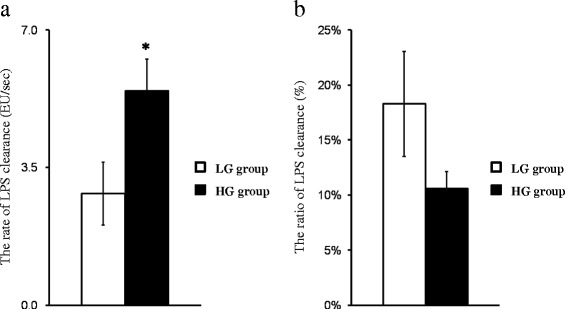
**Effect of feeding high grain diet on LPS clearance in the liver. (a)** The mean rate of LPS clearance in the livers of goats fed high grain (HG) diet was significantly increased compared with goats fed low grain (LG) diet (2.83 EU/sec *vs* 5.46 EU/sec, LG group *vs* HG group); **(b)** the mean percentage of hepatic LPS clearance in the HG group was lower than that in the LG group (18.27% *vs* 10.57%, LG group *vs* HG group). White filled bar, LG group (n = 6, Mean ± SEM); black filled bar, HG group (n = 6, Mean ± SEM), *P < 0.05 **P < 0.01 ***P < 0.001.

According to the calculation of net LPS clearance, the mean rate of hepatic LPS clearance in the HG group was faster than that in the LG group (5.46 EU/sec *vs* 2.83 EU/sec; HG group *vs* LG group, *P* = 0.022, Figure 1a). However, feeding the HG diet to goats declined the percentage of hepatic LPS clearance accounting for the total entry of LPS into liver in the HG group compared to the LG group (10.57% *vs* 18.27%; HG group *vs* LG group, Figure 1b). Yet, the data of the individual percentage of LPS clearance were highly variable and the difference did not obtain statistical significance.

### Pro-inflammatory cytokines and APPs in peripheral blood

After 6 weeks feeding, the plasma concentrations of pro-inflammatory cytokines TNF-α and IL-1β were significantly increased in the HG group compared to the LG group (*P* < 0.001 for both). Although the plasma concentration of IL-6 was increased, there was no significant difference between HG and LG groups (*P* = 0.09, Table 5). Time os sampling did not affect the plasma concentrations of these cytokines.

**Table 5:** **The average concentrations of pro-inflammatory cytokines and acute phase proteins in plasma of peripheral blood of goats**

**Item**	**Diet**		**SED** ^**a**^	**Effect,** ***P*** **-value**		
	**LG**	**HG**		**Diet**	**Time**	**Diet × Time**
TNFα (fmol/mL)						
0 h	15.54	52.36	6.98	<0.001	0.681	0.774
4 h	11.51	71.67				
8 h	19.24	53.92				
IL-1B (ng/mL)						
0 h	0.05	0.11	0.02	<0.001	0.089	0.127
4 h	0.06	0.14				
8 h	0.06	0.09				
IL-6 (pg/mL)						
0 h	93.99	107.02	23.81	0.097	0.372	0.717
4 h	120.56	150.95				
8 h	116.58	161.07				
LBP (μg/mL)						
0 h	13.58	42.22	4.23	<0.001	0.612	0.734
4 h	14.50	42.34				
8 h	16.25	37.91				
Hp (μg/mL)						
0 h	79.55	365.58	50.42	0.021	0.477	0.636
4 h	183.39	307.53				
8 h	112.66	350.52				
SAA (μg/mL)						
0 h	57.31	348.56	37.49	0.024	0.726	0.627
4 h	78.18	386.66				
8 h	63.06	324.88				

^a^SED: standard error of difference between treatment and control.

Compared with feeding of the LG diet, HG diet feeding increased the concentration of LBP (*P* < 0.01), Hp (*P* = 0.021) and SAA (*P* = 0.024) in peripheral blood. The effects of time or interactions with diet on APPs were not significant (Table 5).

### Biochemical parameters of liver function in peripheral blood

The measurement of biochemical parameters refer to ALT, AST, ALP, T-PRO, T-BIL and ALB in peripheral blood was used to determine liver functions. The concentrations of ALT (*P* < 0.001), ALP (*P* = 0.002) and T-BIL (*P* = 0.005) in peripheral blood were higher in the goats fed HG diet compared to the goats fed LG diet. However, feeding a HG diet to goats did not affect plasma concentration of AST (*P* = 0.231), T-PRO (*P* = 0.343) and ALB (*P* = 0.438) in the HG group in comparison to the LG group (Table 6). The effect of interactions of diet with time on plasma concentrations of ALT was significant; however, the effects of time or its interactions with diet on plasma concentrations of the other were not significant.

**Table 6:** **The average concentrations of biochemical parameters in plasma of peripheral blood of goats**

**Item**	**Diet**		**SED** ^**a**^	**Effect,** ***P*** **-value**		
	**LG**	**HG**		**Diet**	**Time**	**Diet × Time**
ALT (IU/L)						
0 h	16.96	26.54	3.16	<0.001	0.529	0.031
4 h	16.20	21.20				
8 h	10.66	33.18				
AST (IU/L)						
0 h	46.92	47.20	5.76	0.231	0.881	0.394
4 h	47.88	50.76				
8 h	45.27	43.25				
ALP (IU/L)						
0 h	55.40	74.20	6.75	0.002	0.787	0.131
4 h	54.20	82.60				
8 h	45.68	67.23				
T-PRO (g/L)						
0 h	64.36	63.88	10.87	0.343	0.682	0.530
4 h	72.18	79.14				
8 h	58.37	57.76				
ALB (g/L)						
0 h	19.58	21.37	50.42	0.438	0.504	0.477
4 h	25.13	29.14				
8 h	29.67	27.48				
T-BIL (μmol/L)						
0 h	2.56	3.38	0.86	0.005	0.845	0.278
4 h	2.64	4.79				
8 h	1.68	3.97				

^a^SED: standard error of difference between treatment and control. IU: international unit.

### Expression of mRNAs encoding genes involved in immune response in the liver

**Figure 2:**
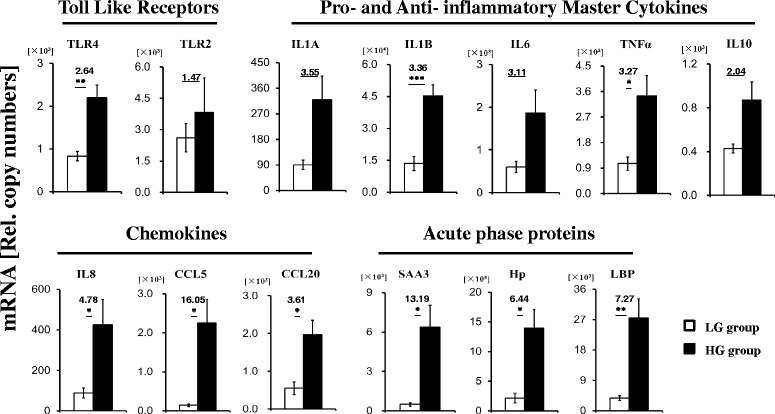
**Quantification of the immune gene expression by RT-qPCR.** Ordinate axis indicate relative copy number (n = 6 in each group, Mean ± SEM). White filled bar: LG group; black filled bar: HG group. Fold changes and significance (*P < 0.05, **P < 0.01, ***P < 0.001) of the selected genes between LG group and HG group are indicated.

Hepatic mRNA expression of 13 different genes involved in immune response, encoding pro- and anti-inflammatory cytokines, chemokines, APPs, TLR4 and TLR2 were assessed. The expression levels of most these genes were up-regulated in the liver of goats fed the HG diet (Figure 2). Expression of TLR4, but not TLR2, was increased (*P* = 0.006) in the liver of goats in the HG group compared to the LG group. A similar fold change in pro-inflammatory cytokine-encoding gene expression in the liver was observed between HG group and LG group (Figure 2). The differences in IL-1β (*P* < 0.001) and TNF-α (*P* = 0.014) gene expression differed between groups, but not for IL-1α (*P* = 0.058) and IL-6 (*P* = 0.074). Expression of IL-10, an anti-inflammatory cytokine produced by dendritic cells, monocytes and particularly macrophages, was 2.04-fold increased, but no significance was observed (*P* = 0.059) in the livers of goats fed different diets. Moreover, feeding with the HG diet significantly enhanced the expression of mRNAs encoding APPs SAA3 (*P* = 0.02), Hp (*P* = 0.023) and LBP (*P* = 0.007), as well as up-regulated the expression of mRNAs encoding key chemokines IL-8 (*P* = 0.042), CCL5 (*P* = 0.02) and CCL20 (*P* = 0.037) in the liver of goats in the HG group compared to the LG group.

### Expression of TLR4 protein in the liver

**Figure 3:**
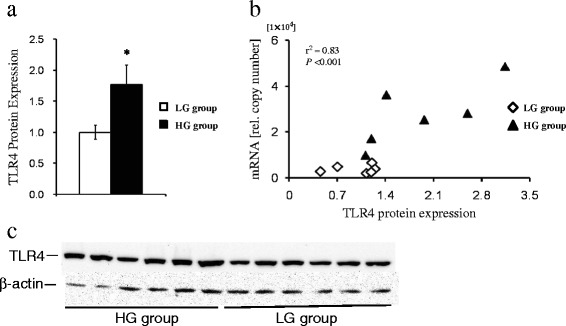
**Effect of feeding high grain diet on expression of TLR4 protein in the liver. (a)** The mean relative protein expression of TLR4 against the reference β-actin protein. White filled bar: LG group (n = 6, Mean ± SEM); black filled bar: HG group (n = 6, Mean ± SEM). Significance (*P < 0.05, **P < 0.01, ***P < 0.001) of TLR4 protein expression is indicated. **(b)** Correlation between TLR4 protein levels and TLR4 mRNA expression. The individual values of TLR4 protein levels were plotted against TLR4 mRNA expression. Rhomboids, LG group; triangles, HG group. r, coefficient of correlation (Pearson); P, significance of correlation. **(c)** Western blotting results of TLR4 and β-actin proteins, HG group (bands 1–6) and LG group (bands 7–12).

Western blotting analyses showed that TLR4 protein expression was enhanced in the liver of goats fed HG diet compared with that in the liver of goats fed LG diet (*P* = 0.043, Figure 3a). In addition, plotting individual relative TLR4 protein expression with the mRNA relative copy numbers revealed a strong correlation (*r* 0.83; *P* < 0.001, Figure 3b).

## Discussion

In recent years, intensive production systems for ruminants have encouraged the use of the HG diet or easily fermentable carbohydrate diet to support high milk yields or rapid weight gain. Although this feeding practice can enhance economic efficiency in the short-term, the feeding of HG diet leads to the translocation of LPS from the digestive system into the circulating blood. In an experiment conducted by replacing 21% of dry matter from a total mixed ration control diet with pellets containing 50% wheat and 50% barley, LPS concentration in the peripheral blood were significantly increased [1]. Other studies showed that feeding a diet containing 60% concentrate to lactating goats or feeding cattle with 70 g grain/kg body weight elevated blood LPS concentrations [28,31]. Moreover, an *in vitro* study by Emmanuel *et al.*, showed five fold increased of the permeability of the rumen wall when this tissue was bathed in pH 5.5 solution, which promoted the translocation of LPS across the rumen wall [10], and Chin *et al.* found that LPS induced cell apoptosis of intestinal epithelial cell lines, disrupted tight junction proteins and enhanced epithelial permeability [35]. *In vivo* studies in which high grain diets were fed to goats caused ruminal and caecal mucosal injury as well as colonic epithelial barrier disruption [4-6]. Taken together, these breaches contribute to the translocation of harmful compounds, such as LPS and histamine, released in the digestive tract into the portal vein occurs during HG diet feeding. In the present study, the feeding of HG diet increased LPS concentrations in circulating blood, especially in the portal vein. In addition, LBP was directly involved in the clearance of LPS in the blood [26,36]. Therefore, an increase of LBP in the peripheral blood of goats fed HG diet support the translocation of LPS from the digestive tract into the bloodstream [1,7]. Alterations of blood flow in response to dietary changes have been reported in previous studies. In a study by Reynolds *et al.*, an increase of dry matter intake significantly increased blood flow in the portal vein and liver of heifers fed 75% alfalfa diet or 75% concentrate diet [37], although the significant difference of blood flow between both diets was not obtained. In present study, the increase of blood flow in the portal vein, hepatic vein and artery indicated that the feeding of HG diet resulted in accelerated blood flow. In addition, it is known that blood flow is affected by body weight, our study observed that the average value of body weight did not change through this experiment. These findings suggest that the feeding of HG diet can promote blood flow entering or exiting the liver, and the increased entry of LPS into the liver through the portal vein. In addition, these findings also demonstrate that the rate of hepatic LPS clearance was significantly increased, but that the percentage of hepatic removed LPS was decreased during HG diet feeding.

There has been increasing attentions on inflammatory responses triggered by HG diet feeding in recent years. Acute phase response (APR) is the early stage of a systemic inflammatory response and was shown in a number of studies investigating the effects of the alterations of the grain proportion in the diet on the health of ruminants. During APR, the concentration of APPs in circulating blood is increased remarkably, and thus, SAA and Hp are often used as inflammatory markers in cattle. Determination of the serum SAA and Hp concentrations demonstrated that Hp but not SAA was notably increased when a diet administered to steers was switched from 0% concentrate diet to 61% concentrate diet [38]. And when this proportion of concentrate was further increased to induce SARA, a rumen metabolic disorder, the serum concentration of SAA was also elevated. These studies confirm another study that also showed that serum concentrations of SAA and Hp were increased when cows received a HG diet [8]. Similar results for serum SAA and Hp levels were observed in a study by Khafipour *et al.*, where LBP plasma concentrations were also increased in this experimental model by increasing the grain percentage of the diet to induce SARA [1]. In the present study, concentrations of SAA, Hp and LBP in peripheral blood were markedly increased, suggesting that feeding the HG diet to goats causes an systemic inflammatory responses.

In addition, we also observed that HG diet feeding elevated plasma concentrations of the pro-inflammatory cytokines TNF-α and IL-1β, but did not affect plasma IL-6 concentrations. The increase in blood pro-inflammatory cytokines also provides evidence for the translocation of LPS and activation of inflammatory responses. The liver is an important immune organ where foreign antigens from the digestive tract encounter the immune system, and immune cells (mononuclear phagocytes) are activated to synthesize or secrete cytokines [39-42]. Thus, during the feeding of HG diet, an increased entry of LPS into the liver via the portal vein probably stimulates Kupffer cells (liver macrophages), the major cytokine-producing liver cells, to release cytokines that subsequently enhance the secretion of APPs from hepatocytes.

Excessive production of cytokines in the liver can damage hepatocytes [22,43]. The biochemical parameters ALT, AST, ALP, T-PRO, ALB and T-BIL in peripheral blood are common indicators used to assess the status of liver function [44]. In particular, ALT is a specific parameter that reflects hepatocyte damage [45]. In the present study, increased plasma concentrations of ALT, ALP and T-BIL suggested that feeding the HG diet resulted in the breach of hepatocytes releasing those parameters into circulation. In previous studies conducted by Scott *et al.*, hepatocytes were shown to be critical for clearance of circulating LPS in the liver [46,47]. Thus, the impairment of hepatocytes during HG diet feeding could contribute to the decreased percentage of LPS clearance in the liver.

There are few studies reporting changes of immune gene expression in the liver in response to variation in diet. The current study showed that HG diet feeding enhanced the expression of many genes involved in immune response, including TLR4, pro- and anti-inflammatory cytokines, chemokines and APPs. Although other hazardous substances in blood and in digesta that can *be translocted* into circulating blood, LPS is thought to be the major contributor to the induction of synthesis of APPs and cytokines in the liver. An early study demonstrated that expression of cytokines and APPs in the liver was up-regulated during LPS intra-mammary gland infusion [12]. The liver is continually exposed to small amounts of LPS translocated from the digestive tract through the mesentery vein directly into the liver via the portal vein [48]. However, the increased concentration of LPS in the portal vein during HG diet feeding in the present study might explain why the enhanced immune gene expression and subsequent inflammatory response in the liver.

Recognition of inflammatory stimuli by the innate immune system is orchestrated by pattern recognition receptors that recognize external stimuli such as pathogen associated molecular patterns (PAMPs) and internal stimuli (damaged associated molecular patterns) [49]. LPS, a well known PAMP, binds to LBP and activates the TLR4-signaling pathway resulting in the production of IL-1β, IL-6 and TNF-α. These pro-inflammatory cytokines play an important role in orchestrating the synthesis of APPs as well as the secretion of other cytokines and chemokines. In our study, the increase of TLR4 and LBP expression in the liver confirmed that the TLR4 signalling pathway was activated by LPS translocated from the digestive tract into the circulating blood. Moreover, the feeding of HG diet elevated the level of mRNAs encoding for the chemokines IL-8, CCL5 and CCL20 in the liver. All these chemokines trigger diapedesis of polymorph nuclear granulocytes, mononuclear cells, macrophages and T-cells through the endothelia of blood vessels into the hepatic inflamed site. Furthermore, expressions of SAA and Hp were also up-regulated in HG group in the present study. Both SAA and Hp have major roles in the innate immune system by opsonising pathogens and removing the potential toxic substances [50].

The general understanding is that a severe local inflammation in peripheral tissues can lead to the release of pro-inflammatory cytokines (IL-1β, IL-6, TNF-α) and anti-inflammatory cytokine IL-10 [51] as well as APPs [52] in the liver. In accordance with previous studies, feeding HG diet to goats resulted in injury to the gastrointestinal barrier and local inflammation [4-6], which might be the cause of the up-regulation of immune genes in the liver. In addition, the reduction of the percentage of hepatic LPS clearance during the feeding of HG diet indicated the accumulation of LPS in systemic circulation. This condition contribute to further explain the conclusion of recent study [53] in which feeding high-starch diets to cows have successful induced subacute ruminal acidosis, which caused the endotoxin tolerance in the mammary gland of cows and suppressed the response of mammary gland to LPS challenge.

## Conclusions

Results obtained in the present study suggest that the feeding of HG diet results in increased blood flow and LPS concentration in the hepatic vein, portal vein and artery, thereby increasing the rate of hepatic LPS clearance, but decreased the percentage of removed LPS accounting for the total entry of LPS into liver. The increase in the rate of hepatic LPS clearance could be due to enhancement of hepatic immune defence response to synthesize and secret cytokines and APPs, and the reduction in the percentage of hepatic LPS clearance might be caused by impaired hepatocytes elicited by the increased entry of LPS into the liver via the portal vein. Overall, our findings provide a fundamental understanding of the effect of the feeding of HG diet on hepatic LPS clearance and immune responses. Future work should focus on the regulation mechanisms of immune relevant gene expression, such as epigenetic modulations, to further elaborate etiology of liver inflammation elicited by the feeding of HG diet.

## Abbreviations

LPS: Lipopolysaccharide
TLR: Toll-like receptor
SARA: Subacute ruminal acidosis
*p*AH: *para*-aminohippuric acid
IL-1α: −1β, −6, Interleukin-1α, 1β, −6
TNF-α: Tumor necrosis factor-α
APR: Acute phase response
APPs: Acute phase proteins
SAA: Serum amyloid A
Hp: Haptoglobin
LBP: Lipopolysaccharide
ALT: Alanine aminotransferase
AST: Aspartate aminotransferase
T-PRO: Total proteins
ALB: Albumin
T-BIL: Total bilirubin
ALP: Alkaline phosphatase
RT-qPCR: Real-time quantitative PCR
